# Fluid therapy is associated with lower care quality and higher symptom burden during last days of life of patients with cancer – a population-based register study

**DOI:** 10.1186/s12904-024-01504-5

**Published:** 2024-07-18

**Authors:** Magnus Lindskog, Hanna Mogensen, Björn Tavelin, Johanna Eknert, Staffan Lundström, Peter Strang

**Affiliations:** 1 Department of Oncology-Pathology, Karolinska Institute, Stockholm, Sweden; 2 Department of Pelvic Cancer, Genitourinary Oncology Unit, Karolinska University Hospital, Eugeniavägen 3, Solna 171 76, Stockholm, Sweden; 3 Department of Immunology, Genetics and Pathology, Cancer Precision Medicine, Uppsala University, Uppsala, Sweden; 4 Unit of Epidemiology, Institute of Environmental Medicine, Karolinska Institutet, Stockholm, Sweden; 5 Department of Radiation Sciences, Umeå University, Umeå, Sweden; 6 Upper GI Unit, Theme Cancer, Karolinska University Hospital, Stockholm, Sweden; 7 Stockholms Sjukhem Foundation and Department of Oncology-Pathology, Stockholm, Sweden

**Keywords:** Fluid therapy, Palliative care, End-of-life, Cancer, Quality, Symptoms

## Abstract

**Background:**

Parenteral fluid (PF) therapy of patients in end-of-life (EOL) is controversial. The purpose of this study was to assess associations between PF, quality of the EOL care process and symptom burden in dying cancer patients, using a population-based approach.

**Methods:**

This was a nationwide retrospective register study of all adult cancer deaths with documented information on PF in the last 24 h of life as reported to the Swedish Register of Palliative Care during a three-year period (*n* = 41,709). Prevalence and relief of symptoms during the last week of life as well as EOL care process quality indicators were assessed in relation to PF in those patients who had a documented decision to focus on EOL care (immediately dying, *n* = 23,112). Odds ratios were calculated, adjusting for place of death (hospital vs. non-hospital).

**Results:**

PF was administered to 30.9% of immediately dying patients in hospitals compared to 6.5% outside of hospitals. PF was associated with a higher likelihood for breathlessness and nausea. In patients screened for EOL symptoms with a validated instrument, PF was inversely associated with the likelihood of complete relief of breathlessness, respiratory secretions, anxiety, nausea and pain. Several palliative care quality indicators were inversely associated with PF, including EOL conversations and prescriptions of injectable drugs as needed. These associations were more pronounced in hospitals.

**Conclusions:**

Parenteral fluid therapy in the last 24 h of life was associated with inferior quality of the EOL care process and with increased symptom burden in imminently dying cancer patients.

**Supplementary Information:**

The online version contains supplementary material available at 10.1186/s12904-024-01504-5.

## Background

Cancer remains one of the leading causes of death worldwide. In order to provide optimal end-of-life (EOL) care, knowledge of common symptoms in dying patients is needed. According to a review by Kehl et al., the four most common symptoms in a general population registered during the last two weeks of life were breathlessness (57%), pain (52%), respiratory secretions/death rattle (51%), and confusion (50%) [1], although the presence of symptoms vary with the type of cancer [2, 3]. Notably, respiratory symptoms and oedema also tend to increase during the last week of life [2]. Diminished oral intake often starts earlier, is gradual and becomes more pronounced as the patients is increasingly bed-bound with fewer and shorter periods of awake time.

Parenteral fluid (PF) therapy in the imminently dying is controversial and whereas patients and families generally are positive, the view among staff varies, with 12-88% of cancer patients receiving PF during their last week of life [4, 5]. It may have a role in a few selected patients who are distressed from severe dehydration, e.g., have symptoms suspected to be caused by hypercalcaemia, or suffer from severe confusion or terminal restlessness, although there is a general lack of evidence to support PF in the last days of life [6, 7]. On the other hand, PF may prolong suffering and lead to unnecessary medicalization of the natural dying process, despite not ameliorating, or, possibly even aggravating EOL symptoms [8–10].

While PF increases the risk of fluid retention, resulting in oedema and worsening of dyspnoea in patients with end-stage renal or heart failure, the effects may vary from beneficial to detrimental in severely ill cancer patients depending on the clinical picture [11–13]. Upon clinical deterioration, a decision to withhold or continue PF in patients who previously benefitted from PF support will pose medical and ethical dilemmas [9]. A sudden decline in general condition is not uncommon in patients with end-stage cancer and may lead to acute hospitalization with exhaustive EOL care including PF that could hamper optimal comfort care and a peaceful death [14].

In this population-based register study, we aimed to describe cancer patients receiving PF in the last 24 h of life with regard to EOL care quality and the prevalence of non-cognitive symptoms, and compare these to cancer patients that did not receive PF at end-of-life.

## Methods

This was a retrospective observational study based on information from the Swedish Registry of Palliative Care (SRPC), which is a nationwide quality register that has been previously described [15]. In short, the register includes individual information regarding EOL care and symptoms in the last week of life for patients dying in hospitals and other care settings in Sweden. The information is extracted from the medical records using a 27-item questionnaire (Q; Supplementary Table S1) and registered retrospectively online by a staff member involved in the EOL care of the particular patient. During the period of the present study, the national coverage for the SRPC was 77% in 2011, 85% in 2012 and 87% in 2013, among patients dying from cancer.

The SRPC is linked with the National Cause of Death Register [16].

### Population

All adult individuals (≥ 18) dying from cancer as the main cause of death, according to data from the National Cause of Death Register, reported to the SRPC between Jan 1, 2011 and Dec 31, 2013 constituted the base of this study (*n* = 41,729). The use of parenteral fluids/nutrition or enteral tube feeding (PF) during the last 24 h of life was assessed with Q19: ‘*Did the person receive parenteral fluids/nutrition or enteraltube feeding during the last 24 hours of life?*

Information regarding symptom prevalence and relief, EOL care decisions, and place of death was obtained from the SRPC and limited to patients whose medical records contained a documented decision by a physician to focus on EOL comfort care (Q 11 A, Table S1), stratifying for deaths in or outside hospitals [17].

### Variables

The following aspects of the quality of the EOL care process (Table S2) were considered: (i) Information to the patient and family about the transition to EOL care; (ii) The use of established instruments to screen for distressing symptoms during the last week of life; (iii) Prescriptions of injectable drugs as needed against common EOL symptoms (pain, anxiety, breathlessness, pulmonary secretions, nausea); (iv) Oral health assessment during the last week of life; and (v) Whether the patient died alone or not. All indicators were dichotomized by implementation or not.

Information on the prevalence (yes/no) of the following documented symptoms during the last week of life was retrieved from the SRPC based on the answer to Q20, Table S1: breathlessness, pulmonary secretions, anxiety, nausea, and breakthrough pain. Analysis of symptom relief was restricted to patients who had their symptoms screened using a validated instrument (Q21 or Q23; Table S1).

### Statistical methods

Patients with and without PF in the last 24 h of life were described with regard to patient demographics and place of death.

The prevalence and relief of symptoms in the last week of life were described in patients with and without PF, and the associations were quantified using odds ratios (OR) with 95% confidence intervals (CI) obtained by Chi2 test for hospitalized and non-hospitalized patients, separately. Patients for whom data was missing about the respective care quality indicator or about a particular symptom were excluded from that specific analysis.

We assessed whether the use of PF in the last 24 h of life was associated with any of the five EOL care quality indicators using logistic regression, restricting the study population to patients with a documented decision by a physician to focus on end-of-life comfort care (Q11A answered by ‘yes’), stratifying the analyses by hospital deaths and non-hospital deaths [17]. Only patients with valid data on PF use (Q19 answered by ‘yes’ or ‘no’, 90% of the patients) were included whereas the 10% with answer ‘unknown’ were excluded from the analyses. In all models the use of PF in the last 24 hr of life were considered as the independent variable. Analyses were conducted using IBM SPSS Statistics for Windows, Version 23.0. Armonk, NY: IBM Corp.

## Results

### Study population

In total, 41,709 patients matching the inclusion criteria were identified (study base) of whom 5,781 (13.9%) received PF in the last 24 h of life. The patient characteristics in relation to PF are described in Table 1. Hospitalized patients were more likely to receive PF (35%) compared to patients dying outside hospital (6%; *p* < 0.0001). Patients with head and neck cancer, esophageal or gastric cancer, ovarian cancer or haematological malignancies were more likely than the average patient to receive PF.

**Table 1:** Study base

Category	ALL	PF	non-PF
**Number of patients**, n (%)	41,709	5,781 (13.9)	35,928 (86.1)
**Age in years**, median (range)	74 (18–105)	70 (18–102)	74 (18–105)
**Place of death**			
Hospital, n (%) Spec PC in-patient unit, n (%) Spec PC home care, n (%) Gen PC home care, n (%) Nursing home short term stay, n (%) Nursing home permanent stay, n (%) Other, n (%)	10,357 13,136 7,402 2,813 4,635 3,189 177	3,627 (35.0) 1,256 (9.6) 502 (6.8) 135 (4.8) 167 (3.6) 74 (2.3) 20 (11.3)	6,730 (65.0) 11,880 (90.4) 6,900 (93.2) 2,678 (95.2) 4,468 (96.4) 3,115 (97.7) 157 (88.7)
**Cancer diagnoses (ICD-10) with PF use > median**			
Head and neck cancer^1^, n (%) Esophageal cancer^2^, n (%) Ovarian cancer^3^, n (%) Hematological cancer^4^, n (%) Gastric cancer^5^, n (%)	474 (100) 786 (100) 1,203 (100) 2,633 (100) 1,488 (100)	146 (30.8) 218 (27.7) 251 (20.1) 517 (19.6) 283 (19.0)	328 (69.2) 568 (72.3) 952 (79.1) 2,116 (80.4) 1,205 (81.0)

PF = Parenteral fluid therapy in the last 24 h of life. ^1^C0-C14, C32-C33; ^2^C15; ^3^C56.9; ^4^C81-C88, C90-C96; ^5^C16

### Associations between PF and quality of the EOL care process

To study potential associations between PF and symptom prevalence and relief, and between PF and quality of the EOL care process in imminently dying patients, we identified patients with a documented decision by a physician to focus on EOL comfort care, *n* = 23,112 (55% of the total study population). Among these, 12% received PF during the last 24 h of life, more commonly in hospitals (30.9%) compared to other places of death (6.5%, *p* < 0.001). Table 2 shows the characteristics of the study subjects including symptom prevalence during the last week of life. Among patients dying outside of hospitals, those who did receive PF were more likely to be cared for in specialized palliative care units (home-based or inpatient) than to die without specialized palliative care (nursing homes or general PC). PF patients were significantly younger, but the absolute age difference was small. Breathlessness and nausea were significantly more common in PF patients, both in the hospital setting and outside of hospitals (Table 2).

**Table 2:** Characteristics of adult cancer patients dying in hospital versus dying outside hospital as reported to the SRPC in 2011–2013

	Hospital deaths PF	Hospital deaths Non-PF	OR	95% CI	*P*	Non-Hospital deaths PF	Non-Hospital deaths Non-PF	OR	95% CI	*P*
N* (%) Age (SD) Sex, % female Received specialised palliative care (%) Pain^#^ Yes/No (% yes) Anxiety^#^ Yes/No (% yes) Nausea^#^ Yes/No (% yes) Breathlessness^#^ Yes/No (% yes) Respiratory secretions^#^ Yes/No (% yes)	1687 (30.9) 70.5 (12.17) 48.9 NA 1279/327 (75.8) 787/593 (46.7) 420/991 (24.9) 604/952 (35.8) 920/714 (54.5)	3772 (69.1) 72.2 (11.99) 49.5 NA 3052/640 (80.9) 1974/1328 (52.3) 685/2618 (18.2) 1065/2485 (28.2) 2029/1674 (53.8)	NA NA 0.98 NA 0.82 0.89 1.62 1.48 1.02	NA NA 0.87–1.10 NA 0.71–0.95 0.79–1.01 1.41–1.87 1.31–1.68 0.95–1.20	NA < 0.0001 0.69 NA 0.009 0.081 < 0.0001 < 0.0001 0.31	1148 (6.5) 68.7 (12.63) 51.4 955 (83.2) 955/181 (83.2) 613/445 (53.4) 429/675 (37.4) 329/785 (28.7) 623/517 (54.3)	16,505 (93.5) 73.9 (12.18) 49.5 10,985 (66.6) 13,655/2744 (82.7) 8565/7108 (51.9) 3633/12,279 (22.0) 3273/12,927 (19.8) 8331/8039 (50.5)	NA NA 1.08 NA 1.06 1.14 2.15 1.66 1.16	NA NA 0.96–1.22 NA 0.90–1.25 1.01–1.30 1.89–2.44 1.45–1.89 1.03–1.31	NA < 0.0001 0.21 < 0.0001 0.48 0.037 < 0.0001 < 0.0001 0.014

PF = parenteral fluid therapy during the last 24 h of life. ^*^Only patients with a documented decision to focus on end-of-life care and with documented PF status (yes or no) were included (*N* = 23,112). ^#^For each specific symptom, only patients for which a documented presence or absence (yes or no) of that symptom were included in the analysis

Table 3 shows the associations of PF and five palliative care process quality indicators in hospitals and non-hospital settings. PF was inversely associated with several of the indicators. Associations were particularly pronounced for hospitalized patients. Compared to hospitalized patients not receiving PF, patients in hospital receiving PF were less frequently prescribed injectable sedatives (81% vs. 90%), antimuscarinics (73% vs. 86%), and antiemetics (63% vs. 70%). Similar but less pronounced associations were noted for patients dying outside hospitals (Table 3). PF patients dying in hospitals were less likely to have an EOL conversation with a physician compared to non-PF patients (68% vs. 79%), and the same pattern was seen for information to the families, although less pronounced. No corresponding differences with respect to EOL conversations were seen for patients dying outside hospitals. Oral status was less likely to be assessed for patients receiving PF compared to non-PF patients (71% vs. 78%). Symptom screening using validated instruments was generally low and did not differ according to PF status. Nor did the risk of dying alone (Table 3).

**Table 3:** Quality indicators of the care process in patients with or without parenteral fluid therapy*, stratified by place of death

	Hospital deaths					Non-hospital deaths				
	PF	Non-PF	OR	95% CI	p	PF	Non-PF	OR	95% CI	p
**EOL conversation, patients**, yes/no (%^¤^)	886/420 (68)	2464/641 (79)	0.55	0.47–0.63	< 0.001	948/119 (89)	13655/1604 (89)	0.94	0.77–1.14	0.51
**EOL conversation, family**, yes/no (%^¤^)	1351/148 (90)	3232/232 (93)	0.66	0.53–0.81	< 0.001	1020/66 (94)	15638/845 (95)	0.89	0.69–1.15	0.39
**Symptom screening**										
Pain, yes/no (%^¤^)	359/1073 (25)	878/2499 (26)	0.95	0.93–1.10	0.50	486/601 (45)	7084/8790 (45)	0.99	0.88–1.13	1.00
Other symptoms, yes/no (%^¤^)	136/1171 (10)	3662792 (12)	0.89	0.72–1.09	0.25	260/789 (25)	3824/11787 (25)	1.02	0.88–1.17	0.83
**Analgesic**^#^, yes/no (%^¤^)	1585/95 (94)	3644/121 (97)	0.55	0.42–0.73	< 0.001	1122/25 (98)	16258/233 (99)	0.64	0.42–0.98	0.04
**Sedative**^#^, yes/no (%^¤^)	1351/308 (81)	3368/369 (90)	0.48	0.41–0.57	< 0.001	1088/59 (95)	15902/503 (97)	0.68	0.51–0.89	0.01
**Antiemetic**^#^, yes/no (%^¤^)	1042/599 (63)	2565/1123 (70)	0.76	0.67–0.86	< 0.001	1053/92 (92)	14997/1464 (91)	1.10	0.90–1.39	0.32
**Antimuscarinic**^#^, yes/no (%^¤^)	1215/439 (73)	3223/512 (86)	0.44	0.38–0.51	< 0.001	1047/98 (91)	15750/734 (96)	0.50	0.40–0.62	< 0.001
**Oral health assessed**, yes/no (%^¤^)	931/388 (71)	2493/700 (78)	0.67	0.58–0.78	< 0.001	831/226 (79)	12468/2892 (81)	0.85	0.73–0.99	0.04
**Died alone**, yes/no (%^¤^)	239/1437 (14)	563/3150 (15)	0.94	0.80–1.10	0.44	129/1015 (11)	1801/14641 (11)	1.03	0.85–1.25	0.74

PF = parenteral fluid therapy in the last 24 hours of life. OR = odds ratio with ’non-PF’ as reference. CI = confidence intervals *Only patients with a documented decision to focus on end-of-life care included (*N* = 23,112); ^¤^% refers to the proportion of “yes”, for each comparison. ^#^Prescription in medical chart of injectable drug to be used as needed. Patients for whom data was missing about the respective care quality indicator were excluded from that specific analysis. The proportion of missing data per care quality indicator were 10% (information to patients), 2.5% (information to families), 6% (screening for pain), 9% (screening for symptoms other than pain), 0% (analgesic), 1% (sedative), 1% (antiemetic), 0% (antimuscarinic), 9.5% (oral health assessment) and 0.5% (died alone)

Finally, we analysed the proportions of patients who achieved complete symptom relief, restricting the analysis to patients who had been screened for symptoms with validated instruments. PF use was inversely associated with the chance of complete symptom relief for all five symptoms assessed (Table 4).

**Table 4:** Likelihood of complete symptom relief^#^ in relation to parenteral fluid therapy in the last 24 h of life

Symptom completely relived	PF	non-PF	OR*	95% CI	*p*
Pain ^#^, yes / no (%^¤^) Anxiety ^#^, yes / no (%^¤^) Nausea ^#^, yes / no (%^¤^) Breathlessness ^#^, yes / no (%^¤^) Respiratory secretions ^#^, yes / no (%^¤^)	2,792 / 1,621 (63) 1,443 / 1,327 (52) 649 / 954 (41) 558 / 1,332 (30) 960 / 2,045 (32)	21,606 / 7,440 (74) 11,517 / 6,421 (64) 4,216 / 3,348 (56) 3,167 / 4,454 (42) 8,412 / 9,565 (47)	0.70 0.73 0.58 0.73 0.66	0.66–0.76 0.67–0.80 0.51–0.65 0.65–0.82 0.61–0.72	<0.001 <0.001 <0.001 <0.001 <0.001

^#^Only patients with a documented symptom screen detecting the specific symptom are included. PF = parenteral fluid therapy; ^¤^% refers to the proportion of “yes”, for each comparison; *OR = odds ratio with ’no PF’ as reference, adjusted for place of death. CI = confidence interval

## Discussion

Parenteral fluid therapy in terminally ill patients remains controversial. Here we report that every three in ten patients immediately dying from cancer in Swedish hospitals received PF in the last 24 h of life which was associated with a lower quality of EOL care. PF was less common in deaths outside of hospitals (nursing homes, specialized or general palliative care). Furthermore, we show that EOL PF is associated with a higher likelihood of breathlessness and nausea and with a lower chance of achieving complete symptom relief.

Patients who received PF in EOL were somewhat younger which is not unexpected. Among the minority of patients dying outside of hospitals who did receive PF (6.5%), we found an association with specialized palliative care. This likely reflects a high threshold to PF in nursing home patients. It might also reflect a perceived benefit of hydration among palliative care physicians to alleviate terminal delirium. For example, in their observational study, Bruera et al. found that using hydration as a component of routine care for patients dying with delirium was associated with a reduction in the incidence of agitated delirium from 26 to 10% in a specialized palliative care unit. The appropriate use of hydration in such situations inevitably requires in depth knowledge in palliative care as well as clinical experience, likely explaining the very low prevalence of PF use found among nursing home residents in the present study [18].

Symptom prevalence in the present study fell within what has previously been published [1–3]. However, the reported variation is large and EOL symptom prevalence could be influenced by variations in study design and sample, as well as in symptom definition and detection methods, as comprehensively shown by Solano et al. in their systematic review [3].

Our approach does not permit causation of identified associations, in particular since symptoms and care quality indicators were registered in the SRPC for the last week of life whereas PF was reported only for the last 24 h. For example, the association of PF and nausea, which was a robust finding across EOL care types, could go in either direction: The threshold to initiate or maintain hydration could be lower in patients with nausea. A plausible example would be hypercalcaemic patients. Or vice versa, PF could lead to volume or nutrient overload, increasing the risk of developing nausea. With respect to breathlessness, however, we find it more likely that EOL PF increases the risk of breathlessness. Although this remains a hypothesis, previous data exist to support it. In their study, Fritzson et al., using matched controls, reported more breathlessness in patients with different diagnoses receiving PF in their last 24 h of life [19]. Moreover, they found an association between increased volume load and breathlessness. In contrast, a prospective single-armed study of 160 cancer patients in their last weeks of life demonstrated that guideline-based fluid therapy may not necessarily be detrimental and that patients with delirium may even benefit from hydration with larger volumes. Most other symptoms did however not improve with larger fluid volumes compared to smaller volume and respiratory secretions were more common with larger fluid volume. Notably there was no control group [20]. A randomised double-blind placebo-controlled trial in terminally ill cancer patients found no improvement in symptoms associated with dehydration, nor with QoL, in response to moderate hydration (1 L) with parenteral saline. The authors discussed the possibility that the substituted volume might have been too small to have a significant positive impact [21]. In the present study, we were limited by the lack of data on fluid volumes. Based on our findings and corroborated by most previously reported data we, however, consider it plausible that PF may aggravate breathlessness in imminently dying patients. Indeed, breathlessness may be difficult to adequately control with limited treatment options. Whereas we found a complete relief of breathlessness in 42% of patients in the non-PF group, only 30% achieved complete relief among PF patients. In addition, our finding of an inverse association of PF and complete relief of other, non-pulmonary, EOL symptoms as well should also be noted.

We analysed five items of the register’s EOL questionnaire that reflect different aspects of the care process and assessed their relations to PF use. In hospitals, EOL discussions were less likely to occur in patients receiving PF despite that the death was anticipated and prepared for by the staff (as seen by a documented EOL care decision). Notably, there was no similar association in patients receiving non-hospital EOL care, possibly indicating that PF is less often considered life-prolonging outside hospitals. However, our findings identify other signs of ambivalence, also outside of hospitals, for dying patient receiving PF, e.g. with respect to PRN prescriptions or assessment of oral health (Table 3). The inverse association of oral health assessment and PF may seem counterintuitive, since signs of dehydration could be evident from a dry oral mucosa. On the other hand, however, an overly active EOL care, that includes parenteral fluid support and is more likely to occur in hospitals, could reflect a continued focus on life-sustaining rather than symptom relieving actions. This would then precipitate for failure to fully and consequently shift to comfort care as well as a to failure to prioritize a necessary EOL discussion, in particular so among staff not trained in palliative medicine [22]. This could also increase the risk of omitting tasks ‘mearly’ related to the patients’ well-being e.g. assessing the oral status. These assumptions are in line with our previous findings from hospital cancer deaths in Sweden [17]. Prescriptions of injectable drugs for symptom control were more likely to be missed in patients receiving PF despite a documented EOL care decision, irrespectively of place of death. This finding is important, however, large scale data analysis, such as the one of the present study, inevitably results in a high sensitivity for detecting small differences that may be statistically significant but not necessarily clinically meaningful. For example, our finding of a 94% vs. 97% prescription rate of injectable analgesics as needed likely does not reflect a clinically meaningful difference. Other larger differences found, however, are more likely to be of clinical significance, e.g. the prescription rates of sedatives or antimuscarinics in hospitalised patients with or without PF, respectively (Table 3).

From these findings, we conclude that an inverse association of PF use and EOL care quality was present in our cohort. These associations were particularly pronounced for hospitalized patients and could reflect a less developed palliative care process in acute hospital wards compared to specialised palliative care units and nursing homes [17]. One possibility is that inconsistencies in the EOL care planning resulted in continued PF use, or potentially vice versa that continued use of PF in EOL led to ambivalence and uncertainty among nurses and physicians about the goal of care.

To our knowledge, our study is the largest population-based investigation into EOL fluid therapy in cancer patients so far published. The study was based on the SRPC which has a high coverage and information is collected coherently. We stratified our results for place of death since the practice of providing PF in EOL differs in hospitals and non-hospital care settings. We have also previously reported higher prevalence of several EOL symptoms in cancer patients dying in hospitals compared to other places of death [17].

The main reason for comparing deaths in hospitals with others is that deaths in emergency hospitals are not expected in the same way as deaths in nursing homes or at palliative care units. For this reason, a higher proportion of PF is expected in hospitals. Internationally, about half of all deaths occur in hospitals [23], which is much higher than in Sweden. With respect to data on PF for dying persons with cancer, most controlled studies are performed in inpatient care according to Cochrane [7], therefore more studies are needed as regards other places of death. Notably, in the SRPC, specialized palliative care inpatient units are not classified as hospitals.

Limitations to our study include the retrospective design and, importantly, the lack of patient-reported outcome measures. The SRPC does not differ between clear fluids or parenteral nutrition and lacks data on the volumes of PF and oral intake. The reporting to the register is done retrospectively and reporting errors are possible, it is also possible that the accuracy of the reporting differs between units. Importantly, the study design only permits associations and precludes conclusions on cause-and-effect.

In our cohort, the prevalence of symptom screening with validated instrument in the last week of life was low, independently from PF, especially in hospitals, 25–45% for pain and 10–25% for other symptoms. This however agrees relatively well with the findings of Jordhøy et al. who found symptom screening completeness to fall significantly to 25–36% within the last 30 days before death [24]. In a study by de la Cruz et al., on EOL symptom prevalence among cancer patients included in an RCT on parenteral hydration and receiving hospice care, 30% of all randomized patients were identified to have undergone symptom assessment during the last week of life [25]. Terminal delirium is relatively common in EOL which would be expected to pose challenges to staff to reliably screen for specific symptoms, in particular outside of specialized palliative care. Even in patients who maintain lucid in EOL, drowsiness is prevalent. Data on confusion was not extracted from the SRPC in this study. However, we aimed to focus on non-cognitive symptoms since the potential benefits of PF in delirium have been previously investigated [18, 26, 27]. The fact that the SRPC registers symptoms and care quality indicators during the last week of life, while the use of PF is reported for the last 24 h, is an important limitation that obscures the understanding of potential cause-and-effect in our study, as discussed above. Hence, we cannot rule out that PF in some patients might have been initialised as a response to distressing EOL symptoms in an attempt to alleviate them.

Our cohort consisted of patients dying from cancer reported to the SRPC in 2011–2013. It is possible that the attitude towards employing PF in end-of-life may have changed since then because of increased awareness of potential downsides of PF. However, using the same questionnaire during the COVID-19 pandemic in a study comparing those nursing home residents who died in their nursing home (*n* = 1903) with those who were acutely admitted to and died in acute hospitals (*n* = 202), the percentage of PF was 6% in nursing homes versus 38% in hospitals [28], i.e., figures close to the data in the current study. This indicates that the use of EOL PF in hospitalized patients does not seem to have changed significantly with time over the last ten years in Sweden. Nineteen per cent of all cancer deaths in Sweden during the study period were not reported to the SRPC and we cannot know if the association between PF use and quality of EOL care or symptom prevalence in that group differs from the associations found in this study.

## Conclusions

This large nationwide register study provides evidence of associations of end-of-life parental fluid therapy with worse symptom control, inconsistent care and inferior quality of dying for cancer patients in Sweden.

### Electronic supplementary material

Below is the link to the electronic supplementary material.

Supplementary Material 1

## Data Availability

The datasets used and/or analysed during the current study are available from the corresponding author on reasonable request.

## Abbreviations

PF: Parenteral fluid therapy
EOL: End-of-life
SRPC: The Swedish Registry of Palliative Care

## References

[1] Kehl KA, Kowalkowski JA. A systematic review of the prevalence of signs of impending death and symptoms in the last 2 weeks of life. Am J Hosp Palliat Care. 2013;30(6):601–16. 23236090 10.1177/1049909112468222

[2] Hiratsuka Y, Suh SY, Won SH, Kim SH, Yoon SJ, Koh SJ, Kwon JH, Park J, Ahn HY, Cheng SY, et al. Prevalence and severity of symptoms and signs in patients with advanced cancer in the last days of life: the east Asian collaborative cross-cultural study to elucidate the dying process (EASED). Support Care Cancer. 2022;30(6):5499–508. 35304634 10.1007/s00520-022-06969-9

[3] Solano JP, Gomes B, Higginson IJ. A comparison of symptom prevalence in far advanced cancer, AIDS, heart disease, chronic obstructive pulmonary disease and renal disease. J Pain Symptom Manage. 2006;31(1):58–69. 16442483 10.1016/j.jpainsymman.2005.06.007

[4] Morita T, Shima Y, Adachi I, Japan Palliative Oncology Study G. Attitudes of Japanese physicians toward terminal dehydration: a nationwide survey. J Clin Oncol. 2002;20(24):4699–704. 12488416 10.1200/JCO.2003.10.155

[5] Raijmakers NJH, van Zuylen L, Costantini M, Caraceni A, Clark J, Lundquist G, Voltz R, Ellershaw JE, van der Heide A. Opcare: Artificial nutrition and hydration in the last week of life in cancer patients. A systematic literature review of practices and effects. Ann Oncol. 2011;22(7):1478–86. 21199887 10.1093/annonc/mdq620

[6] Hui D, Dev R, Bruera E. The last days of life: symptom burden and impact on nutrition and hydration in cancer patients. Curr Opin Support Palliat Care. 2015;9(4):346–54. 26509860 10.1097/SPC.0000000000000171 PMC4792116

[7] Buchan EJ, Haywood A, Syrmis W, Good P. Medically assisted hydration for adults receiving palliative care. Cochrane Database Syst Rev. 2023;12(12):Cd006273. 38095590 10.1002/14651858.CD006273.pub4 PMC10720602

[8] Dev R, Dalal S, Bruera E. Is there a role for parenteral nutrition or hydration at the end of life? Curr Opin Support Palliat Care. 2012;6(3):365–70. 22801468 10.1097/SPC.0b013e328356ab4a

[9] Druml C, Ballmer PE, Druml W, Oehmichen F, Shenkin A, Singer P, Soeters P, Weimann A, Bischoff SC. ESPEN guideline on ethical aspects of artificial nutrition and hydration. Clin Nutr. 2016;35(3):545–56. 26923519 10.1016/j.clnu.2016.02.006

[10] Good P, Richard R, Syrmis W, Jenkins-Marsh S, Stephens J. Medically assisted hydration for adult palliative care patients. Cochrane Database Syst Rev. 2014;2014(4):CD006273. 24760678 10.1002/14651858.CD006273.pub3 PMC8988261

[11] Morita T, Hyodo I, Yoshimi T, Ikenaga M, Tamura Y, Yoshizawa A, Shimada A, Akechi T, Miyashita M, Adachi I, et al. Association between hydration volume and symptoms in terminally ill cancer patients with abdominal malignancies. Ann Oncol. 2005;16(4):640–7. 15684225 10.1093/annonc/mdi121

[12] Bozzetti F, Arends J, Lundholm K, Micklewright A, Zurcher G, Muscaritoli M. Espen: ESPEN guidelines on Parenteral Nutrition: non-surgical oncology. Clin Nutr. 2009;28(4):445–54. 19477052 10.1016/j.clnu.2009.04.011

[13] Arends J, Bachmann P, Baracos V, Barthelemy N, Bertz H, Bozzetti F, Fearon K, Hutterer E, Isenring E, Kaasa S, et al. ESPEN guidelines on nutrition in cancer patients. Clin Nutr. 2017;36(1):11–48. 27637832 10.1016/j.clnu.2016.07.015

[14] McCann RM, Hall WJ, Groth-Juncker A. Comfort care for terminally ill patients. The appropriate use of nutrition and hydration. JAMA. 1994;272(16):1263–6. 7523740 10.1001/jama.1994.03520160047041

[15] Lundstrom S, Axelsson B, Heedman PA, Fransson G, Furst CJ. Developing a national quality register in end-of-life care: the Swedish experience. Palliat Med. 2012;26(4):313–21. 21737480 10.1177/0269216311414758

[16] Brooke HL, Talback M, Hornblad J, Johansson LA, Ludvigsson JF, Druid H, Feychting M, Ljung R. The Swedish cause of death register. Eur J Epidemiol. 2017;32(9):765–73. 28983736 10.1007/s10654-017-0316-1 PMC5662659

[17] Elmstedt S, Mogensen H, Hallmans DE, Tavelin B, Lundstrom S, Lindskog M. Cancer patients hospitalised in the last week of life risk insufficient care quality - a population-based study from the Swedish Register of Palliative Care. Acta Oncol. 2019;58(4):432–8. 30633611 10.1080/0284186X.2018.1556802

[18] Bruera E, Franco JJ, Maltoni M, Watanabe S, Suarez-Almazor M. Changing pattern of agitated impaired mental status in patients with advanced cancer: association with cognitive monitoring, hydration, and opioid rotation. J Pain Symptom Manage. 1995;10(4):287–91. 7602179 10.1016/0885-3924(95)00005-J

[19] Fritzson A, Tavelin B, Axelsson B. Association between parenteral fluids and symptoms in hospital end-of-life care: an observational study of 280 patients. BMJ Support Palliat Care. 2015;5(2):160–8. 24644196 10.1136/bmjspcare-2013-000501

[20] Yamaguchi T, Morita T, Shinjo T, Inoue S, Takigawa C, Aruga E, Tani K, Hara T, Tamura Y, Suga A, et al. Effect of parenteral hydration therapy based on the Japanese national clinical guideline on quality of life, discomfort, and symptom intensity in patients with advanced cancer. J Pain Symptom Manage. 2012;43(6):1001–12. 22651946 10.1016/j.jpainsymman.2011.06.028

[21] Bruera E, Hui D, Dalal S, Torres-Vigil I, Trumble J, Roosth J, Krauter S, Strickland C, Unger K, Palmer JL, et al. Parenteral hydration in patients with advanced cancer: a multicenter, double-blind, placebo-controlled randomized trial. J Clin Oncol. 2013;31(1):111–8. 23169523 10.1200/JCO.2012.44.6518 PMC3530688

[22] Piili RP, Lehto JT, Luukkaala T, Hinkka H, Kellokumpu-Lehtinen PI. Does special education in palliative medicine make a difference in end-of-life decision-making? BMC Palliat Care. 2018;17(1):94. 30021586 10.1186/s12904-018-0349-6 PMC6052558

[23] Broad JB, Gott M, Kim H, Boyd M, Chen H, Connolly MJ. Where do people die? An international comparison of the percentage of deaths occurring in hospital and residential aged care settings in 45 populations, using published and available statistics. Int J Public Health. 2013;58(2):257–67. 22892713 10.1007/s00038-012-0394-5

[24] Jordhoy MS, Fayers P, Loge JH, Ahlner-Elmqvist M, Kaasa S. Quality of life in palliative cancer care: results from a cluster randomized trial. J Clin Oncol. 2001;19(18):3884–94. 11559726 10.1200/JCO.2001.19.18.3884

[25] de la Cruz M, Noguera A, San Miguel-Arregui MT, Williams J, Chisholm G, Bruera E. Delirium, agitation, and symptom distress within the final seven days of life among cancer patients receiving hospice care. Palliat Support Care. 2015;13(2):211–6. 24556057 10.1017/S1478951513001144

[26] Bruera E, Sala R, Rico MA, Moyano J, Centeno C, Willey J, Palmer JL. Effects of parenteral hydration in terminally ill cancer patients: a preliminary study. J Clin Oncol. 2005;23(10):2366–71. 15800328 10.1200/JCO.2005.04.069

[27] Lawlor PG, Gagnon B, Mancini IL, Pereira JL, Hanson J, Suarez-Almazor ME, Bruera ED. Occurrence, causes, and outcome of delirium in patients with advanced cancer: a prospective study. Arch Intern Med. 2000;160(6):786–94. 10737278 10.1001/archinte.160.6.786

[28] Strang P, Martinsson L, Bergstrom J, Lundstrom S. COVID-19: symptoms in dying residents of nursing homes and in those admitted to hospitals. J Palliat Med. 2021;24(7):1067–71. 33667124 10.1089/jpm.2020.0688

