# Supplementary material for: Fluid therapy is associated with lower care quality and higher symptom burden during last days of life of patients with cancer – a population-based register study
Source: BMC Palliat Care. 2024 Jul 18;23:178. doi: 10.1186/s12904-024-01504-5 (PMC11256446; doi:10.1186/s12904-024-01504-5)
Supplement: Supplementary file 1 — Supplementary Material 1 [file 12904_2024_1504_MOESM1_ESM.docx]

**Supplementary tables**

**Table S1. English version of the questionnaire used for registering deaths in the Swedish Register of Palliative Care since January 1 2012**

| **No.** | **Question** | **Reply options** |
| --- | --- | --- |
| 1 | Unit identification code |  |
| 2 | Personal identity number of the deceased person |  |
| 3 | First and last name of the deceased person |  |
| 4 | Date of death (year/month/day) |  |
| 5 | Date (year/month/day) when the person was admitted to the unit where the death occurred (for home care, please state the date when home care was initiated) |  |
| 6 | The place of death is best described as: | - Nursing home – permanent stay - Nursing home – short‑term stay - Hospital ward (not hospice/palliative in‑patient care) - Hospice/palliative in‑patient care - Own home with support from specialised home‑care team - Own home with support from general home‑care team - Other, specify |
| 7 | Disease/basic state that caused the death (more than one answer is possible): | - Cancer - Cardiovascular disease - Respiratory disease - Dementia - Stroke - Other neurological disease - Diabetes - State after fracture - Multimorbidity - Other, namely: |
| 8 | Will there be a post‑mortem examination of the deceased? | - Yes, forensic - Yes, regular clinical - No   If the answer is Yes, forensic, only answer questions 28-30. If the answer is No or Yes, regular clinical, go to question 9. |
| 9 | Based on the disease trajectory, was the death expected? | - Yes - No - Don't know   If the answer is Yes or Don't know, answer all the following questions. If the answer is No, answer only questions 14, 16, 18, 28-30. |
| 10 | How long before death did the person lose the ability to express his/her will and take part in decisions concerning the content of medical care? | - Retained ability until end of life - Hour/hours - Day/days - Week/weeks - Month or more - Don't know |
| 11A | Do the medical records include a documented decision by the physician responsible to shift treatment/care to end-of-life care? | - Yes, in free text - Yes, as a classification code - No - Don't know |
| 11B | Did the person receive information about the transition to end-of-life care, i.e. an individually tailored and informed conversation with a physician that is documented in the medical records about being in the final stage of life and about care being focused on quality of life and symptom relief? | - Yes - No - Don't know |
| 12 | Was the place of death in line with the person's last stated wishes? | - Yes - No - Don't know |
| 13A | Did the person have pressure ulcers upon arrival at your unit (specify highest grade occurring)? | - Yes, grade 1 - Yes, grade 2 - Yes, grade 3 - Yes, grade 4 - No - Don't know   If the answer is Yes (grades 1-4), answer question 13B. If the answer is No or Don't know, skip to question 14A. |
| 13B | Were the pressure ulcers documented? | - Yes - No - Don't know |
| 14A | Did the person die with pressure ulcers (specify highest grade occurring)? | - Yes, grade 1 - Yes, grade 2 - Yes, grade 3 - Yes, grade 4 - No - Don't know   If the answer is Yes (grade 1-4), answer question 14B. If the answer is No or Don't know, skip to question 15A. |
| 14B | Were the pressure ulcers documented? | - Yes - No - Don't know |
| 15A | Was the person’s oral health assessed during the last week of life? | - Yes - No - Don't know   If the answer is Yes, answer question 15B. If the answer is No or Don't know, skip to question 16. |
| 15B | Was any disorder noted during assessment? | - Yes - No - Don't know   If the answer is Yes or No, answer question 15C. If the answer is Don't know, skip to question 16. |
| 15C | Was the assessment of oral health documented? | - Yes - No - Don't know |
| 16 | Was anyone present at the time of death? | - Yes, close friend(s) or relative(s) - Yes, close friend(s)/relative(s) and staff - Yes, staff - No - Don't know |
| 17 | Did the person’s close friend(s)/relative(s) receive information about transition to end-of-life care, i.e. an individually tailored and informed conversation with a physician that is documented in the medical records about being in the final stage of life and about care being focused on quality of life and symptom relief? | - Yes - No - Don't know - Had no close friend(s)/relative(s)   If the answer is Yes, No or Don't know, go to question 18. If the answer is Had no close friend(s)/relative(s), skip to question 19. |
| 18 | Was/were the person’s close friend(s)/relative(s) offered a follow-up talk within 1-2 months of the death? | - Yes - No - Don't know |
| 19 | Did the person receive parenteral fluids/nutrition or enteral‑tube feeding during the last 24 hours of life? | - Yes - No - Don't know |
| 20 | Did the person display any of the following symptoms (20A-F) at any time during the last week of life? |  |
| 20A | Pain | - Yes - No - Don't know   If the answer is Yes, answer the following question. If the answer is No or Don't know, skip to question 20B. |
|  | Pain was relieved: | - Completely - Partially - Not at all |
| 20B | Death rattle | - Yes - No - Don't know   If the answer is Yes, answer the following question. If the answer is No or Don't know, skip to question 20C. |
|  | Death rattle was relieved: | - Completely - Partially - Not at all |
| 20C | Nausea | - Yes - No - Don't know   If the answer is Yes, answer the following question. If the answer is No or Don't know, skip to question 20D. |
|  | Nausea was relieved: | - Completely - Partially - Not at all |
| 20D | Anxiety | - Yes - No - Don't know   If the answer is Yes, answer the following question. If the answer is No or Don't know, skip to question 20E. |
|  | Anxiety was relieved: | - Completely - Partially - Not at all |
| 20E | Dyspnoea | - Yes - No - Don't know   If the answer is Yes, answer the following question. If the answer is No or Don't know, skip to question 20F. |
|  | Dyspnoea was relieved: | - Completely - Partially - Not at all |
| 20F | Confusion | - Yes - No - Don't know   If the answer is Yes, answer the following question. If the answer is No or Don't know, skip to question 21. |
|  | Confusion was relieved: | - Completely - Partially - Not at all |
| 21 | Was the person’s pain assessed at any documented time during the last week of life using VAS, NRS or another validated pain-assessment tool? | - Yes - No - Don't know |
| 22 | Did the person experience severe pain at any time during the last week of life (e.g. VAS/NRS > 6 or severe pain according to another validated pain-assessment tool)? | - Yes - No - Don't know |
| 23 | Were the person’s other symptoms assessed at any time during the last week of life using VAS, NRS or another validated symptom-assessment tool? | - Yes - No - Don't know |
| 24 | Was there an individual prescription of injectable PRN drugs on the drug list before death? |  |
|  | Opioids against pain | - Yes - No - Don't know |
|  | Drugs against death rattle | - Yes - No - Don't know |
|  | Drugs against nausea | - Yes - No - Don't know |
|  | Drugs against anxiety | - Yes - No - Don't know |
| 25 | How long before death was the person last examined by a physician? | - Day/days - Week/weeks - Month or more - Don't know |
| 26 | Were specialists outside the team/ward consulted concerning the person’s symptom relief during the last week of life (more than one answer option is possible)? | - Yes, pain clinic - Yes, palliative-care team - Yes, other hospital unit - Yes, social worker/physiotherapist/ occupational therapist/dietician - Yes, spiritual counsellor - No - Don't know |
| 27 | How satisfied is the team with the care delivered to the person during the last week of life? | A 5-point scale ranging from Not at all (1) to Completely (5) |
| 28 | Date (year/month/day) of answering the questions |  |
| 29 | The questionnaire was answered by: | - A single employee - Staff jointly |
| 30 | Name and e-mail address of registrant, occupation | - Physician - Nurse - Other staff |

**Table S2. Items from the end-of-life questionnaire selected as quality indicators of the care process**

| **Question number** | **Question content** | **Theme** | **Type of indicator** |
| --- | --- | --- | --- |
| 11 B  15A  16 | Did the person receive information about the transition to end-of-life care, i.e. an individually tailored and informed conversation with a physician that is documented in the medical records about being in the final stage of life and about care being focused on quality of life and symptom relief?  Was the person’s oral health assessed during the last week of life?  Was anyone present at the time of death | Information  Clinical assessment  Existential support | Care process  Care process  Care process |
| 17 | Did the person’s close friend(s)/relative(s) receive information about transition to end-of-life care, i.e. an individually tailored and informed conversation with a physician that is documented in the medical records about being in the final stage of life and about care being focused on quality of life and symptom relief? | Information | Care process |
| 21 | Was the person’s pain assessed at any documented time during the last week of life using VAS, NRS or another validated pain-assessment tool? | Symptom screening | Care process |
| 23 | Were the person’s other symptoms assessed at any time during the last week of life using VAS, NRS or another validated symptom-assessment tool? | Symptom screening | Care process |
| 24 | Was there an individual prescription of an injectable PRN opioid against pain on the drug list before death? | Symptom control | Care process |
| 24 | Was there an individual prescription of an injectable PRN drug against death rattles on the drug list before death? | Symptom control | Care process |
| 24 | Was there an individual prescription of an injectable PRN drug against nausea on the drug list before death? | Symptom control | Care process |
| 24 | Was there an individual prescription of an injectable PRN drug against anxiety on the drug list before death? | Symptom control | Care process |
